# Spatiotemporal Dynamics of Maize (*Zea mays* L.) Root Growth and Its Potential Consequences for the Assembly of the Rhizosphere Microbiota

**DOI:** 10.3389/fmicb.2021.619499

**Published:** 2021-03-17

**Authors:** Michael Bonkowski, Mika Tarkka, Bahar S. Razavi, Hannes Schmidt, Evgenia Blagodatskaya, Robert Koller, Peng Yu, Claudia Knief, Frank Hochholdinger, Doris Vetterlein

**Affiliations:** ^1^Terrestrial Ecology, Institute of Zoology, University of Cologne, Cologne, Germany; ^2^Department of Soil Ecology, Helmholtz Centre for Environmental Research – UFZ, Halle, Germany; ^3^German Centre for Integrative Biodiversity Research (iDiv) Halle–Jena–Leipzig, Leipzig, Germany; ^4^Department of Soil and Plant Microbiome, Christian-Albrecht University of Kiel, Kiel, Germany; ^5^Centre for Microbiology and Environmental Systems Science, University of Vienna, Vienna, Austria; ^6^Institute of Bio- and Geosciences, IBG-2: Plant Sciences, Forschungszentrum Jülich GmbH, Jülich, Germany; ^7^Emmy Noether Group Root Functional Biology, Institute of Crop Science and Resource Conservation (INRES), University of Bonn, Bonn, Germany; ^8^Institute of Crop Science and Resource Conservation – Molecular Biology of the Rhizosphere, University of Bonn, Bonn, Germany; ^9^Crop Functional Genomics, Institute of Crop Science and Resource Conservation (INRES), University of Bonn, Bonn, Germany; ^10^Department of Soil System Science, Helmholtz Centre for Environmental Research – UFZ, Halle, Germany; ^11^Soil Science, Martin-Luther-University Halle-Wittenberg, Halle, Germany

**Keywords:** rhizosphere, microbiota, protists, community assembly, microbiome, spatiotemporal dynamics, self-organization

## Abstract

Numerous studies have shown that plants selectively recruit microbes from the soil to establish a complex, yet stable and quite predictable microbial community on their roots – their “microbiome.” Microbiome assembly is considered as a key process in the self-organization of root systems. A fundamental question for understanding plant-microbe relationships is where a predictable microbiome is formed along the root axis and through which microbial dynamics the stable formation of a microbiome is challenged. Using maize as a model species for which numerous data on dynamic root traits are available, this mini-review aims to give an integrative overview on the dynamic nature of root growth and its consequences for microbiome assembly based on theoretical considerations from microbial community ecology.

## Introduction

Numerous studies have shown that plants selectively recruit microbes from the soil to establish a complex, yet stable and quite predictable microbial community on their roots – their “microbiome” (Berg and Smalla, 2009; Hartmann et al., 2009; Weinert et al., 2010). Microbiome assembly is considered as a key process in the self-organization of root systems (Vetterlein et al., 2020). Better control of microbiome assembly would improve plant health and fitness by promoting beneficial microbial traits (Friesen et al., 2011; Oyserman et al., 2018; Wille et al., 2019). A fundamental question for understanding plant-microbe relationships is where a predictable microbiome is formed along the root axis and through which microbial dynamics the stable formation of a microbiome is challenged. Theoretically, community assembly begins with random, unregulated colonization of taxa from nearby sites (i.e., neutral processes), a process that continues throughout the lifetime of roots; while ordered dynamics (microbiome assembly) occur through selection (i.e., niche-based processes) when (i) exudates promote fast-growing copiotrophic taxa, (ii) root signals attract specific symbionts or pathogens, (iii) increased competition due to limited resource availability leads to species sorting, and (iv) predation selects for specific microbial traits among members of the microbiome (Vellend, 2010; Hardoim et al., 2011; Ho et al., 2017; Kudjordjie et al., 2019; Amacker et al., 2020; Chen et al., 2020). These microbial assembly processes again are embedded in plant-driven spatiotemporal dynamics at small and large scales, caused by differences in the quality and quantity of rhizodeposition: (i) along the root axis, (ii) during diurnal cycles, (iii) on different root types, and (iv) during plant development. Emphasizing maize as a model species for which numerous data on dynamic root traits are available, this mini-review aims to give an integrative overview on the dynamic nature of root growth and its consequences for microbiome assembly based on theoretical considerations from microbial community ecology.

## The Root System of Maize: a Cereal Model for Dissecting Plant-Microbial Interactions

Maize displays a high degree of genomic diversity (Hake and Ross-Ibarra, 2015) and its specific root system facilitates the extraction of water and mineral nutrients from the soil (Yu et al., 2016b). The maize root system bears different root types and responds sensitively to the availability of nutrients (Yu et al., 2015, 2016a). It is composed of embryonic roots including primary and seminal roots and postembryonic root types such as crown roots, and these different root types influence the form and function of the root system at different developmental stages throughout the whole life cycle of maize plants (Hochholdinger et al., 2018). The different maize root types show specific anatomical properties, distinct gene expression patterns (Tai et al., 2016), and root type specific metabolic signatures that affect both bacterial and fungal communities inhabiting respective root types (Yu et al., 2018; Cotton et al., 2019). For example, benzoxazinoids have an influence on both the rhizosphere microbiome and root herbivory (Kudjordjie et al., 2019) and are particularly enriched in lateral and crown root exudates (Park et al., 2004). Furthermore, plant genotypes in maize, due to domestication and breeding, influence community assembly of microbiota (Bouffaud et al., 2012, 2014; Peiffer et al., 2013; Szoboszlay et al., 2015). The relative strength of plant genotype effect is at its strongest within root tissues, while soil origin has often a more significant impact on rhizosphere communities (Edwards et al., 2015; Bonito et al., 2019; Veach et al., 2019; Brown et al., 2020). Although the optimization of microbial formulas using synthetic communities is advancing, crop breeding programs have not yet incorporated the selection of beneficial traits of rhizosphere microorganisms (Trivedi et al., 2020). The perhaps most promising trait in this respect is pathogen resistance (Zhao et al., 2004; Qiu et al., 2020), where the rhizosphere community has proved helpful (Compant et al., 2005; Hohmann and Messmer, 2017). For instance, a common bean cultivar resistant to *Fusarium oxysporum* selects for a particular rhizosphere microbiome with enriched functional traits related to the first line of defense against the pathogen, biosynthesis machineries for antifungal phenazines, and fungal membrane damaging small molecules, rhamnolipids (Mendes et al., 2018). We refer to Schmidt et al. (2016) for a more thorough discussion of the effects of maize domestication on changes in root traits and possible consequences for microbiome function that may guide future studies. Taken together, maize appears as an ideal model crop species to dissect the relationships between root structure, plant genetic diversity, and breeding history, and their interaction with soil microbes under diverse environmental conditions (Yu and Hochholdinger, 2018).

## Rhizosphere Community Assembly Along the Growing Root Axis

The microbiome concept implies guidance of microbial assembly processes by plant roots, but the spatio-temporal dynamics of root growth requires the concurrent reassembly of microbial communities along the root axis (Kuzyakov and Razavi, 2019).

Microbiome assembly starts when microorganisms first encounter the cap of a maize root, which is actively generated in the very tips of the roots (Hochholdinger, 2009). It consists of border cells that separate from the root and are embedded in a water-soluble polysaccharide mucilage matrix (Hawes et al., 2016). The translocation of recently fixed photosynthates to root tips takes less than 1 h (Henkes et al., 2008; Fischer et al., 2010), but the great majority of soil microorganisms in bulk soil resides in a dormant state (Blagodatskaya and Kuzyakov, 2013). It takes about half a day until bacteria and other fast growing microorganisms have entered an exponential growth phase while root growth continues (Watt et al., 2006b; Bonkowski and Clarholm, 2012). Average root growth in maize was shown to proceed at a rate of 2 cm d^−1^ for primary roots, 0.75 cm d^−1^ for first and second order lateral roots, and 3 cm d^−1^ for the seminal roots and the shoot born crown roots (de Moraes et al., 2019). Because only a part of bulk soil microorganisms (e.g., the fast-growing, “copiotrophic” microorganisms) possess the physiological prerequisites to exploit the transient resource pulses from rhizodeposits (Ho et al., 2017), the rhizosphere microbiota are generally characterized by a lower alpha diversity and evenness compared to bulk soil communities (Gomes et al., 2001; Haichar et al., 2008; Aira et al., 2010; Peiffer et al., 2013; Ofek et al., 2014; Walters et al., 2018).

## Microbiome Assembly From a Microbial Perspective

For the colonization of root tips, community assembly theory assumes highest randomness through priority effects, because species that arrive first (both by active migration and by shifting from dormancy into activity) have a competitive advantage over later arriving species by exploiting (niche preemption) and/or modifying (niche modification) the available resources (Fukami, 2015; Nuccio et al., 2020). Dominance of different taxa on different root tips then should lead to highest variation of microbial beta diversity (Anderson et al., 2006; Rüger et al., 2021).

**Figure 1:**
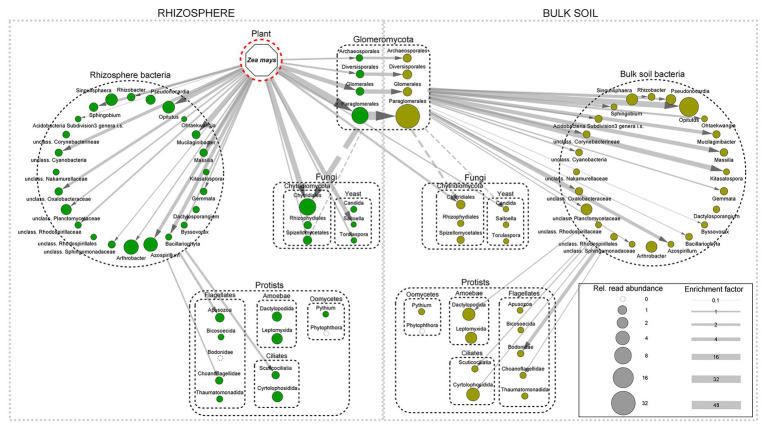
Incorporation of plant-derived carbon (^13^C) into RNA of rhizosphere and bulk soil microbiota 2 days after continuous ^13^CO_2_ labeling over 6 days of 32 day-old maize plants. Rhizosphere taxa are shown in green, bulk soil taxa in brown, and size of circles corresponds to read abundance of enriched taxa. Arrow width indicates the relative magnitude of C-flow based on ^13^C-enrichment factors of the organisms. The depicted C flow only represents the assimilated ^13^C and thus underestimates total C-flow by not taking into account the respired ^13^C at various trophic levels. Dotted arrows indicate potential C flow based on trophic relationships. The C-flow network implemented in Cytoscape based on data of Hünninghaus et al. (2019).

Most important in this respect, but still understudied, are priority effects of root-infecting symbionts, such as mycorrhizae. Plants allocate up to 30% of total photosynthate to mycorrhiza (Bending et al., 2006; Moyano et al., 2007; Brüggemann et al., 2011), and root mycorrhization again strongly feeds back on community assembly of other rhizobiota (Mar Vázquez et al., 2000; Henkes et al., 2018; Sarkar et al., 2019). For example, a recent SIP study by Hünninghaus et al. (2019) demonstrated the central role of mycorrhizal fungi (Glomeromycota) on maize roots (Figure 1) as a carbon shunt to its associated “mycorrhizosphere” microbiome (Nuccio et al., 2013). The trade-offs and multiple reciprocal interactions between root-infecting and free-living microbiota certainly deserve more attention (Andrade et al., 1998; Xavier and Germida, 2003; Frey-Klett and Garbaye, 2005; Koller et al., 2013a,b; Rudnick et al., 2015; Rodríguez-Caballero et al., 2017), but would exceed the scope of this minireview.

In addition, microevolutionary processes among free-living rhizobiota can affect the composition and function of the rhizosphere microbiome (Prosser et al., 2007). Zee and Fukami (2018) showed that priority effects of competing bacterial populations weakened within weeks due to fast sympatric co-evolution, leading to increased niche differentiation and phenotypic complementarity among taxa. It has been assumed that community assembly of free-living rhizobiota should be subject to substantial variability, as plant hosts cannot selectively sanction or punish non-rewarding free-living microorganisms (Denison et al., 2003). In particular, the common plant growth promoting traits of rhizosphere bacteria are based on the production of “public goods” (e.g., exoenzymes for nutrient mineralization, siderophores for nutrient capture, etc.) that are costly to produce for the individual and prone to exploitation by non-cooperating cheaters in microbial communities (Denison et al., 2003; Keller and Surette, 2006; West et al., 2006; Jousset et al., 2009). Bacteria respond to metabolic burdens by fast adaptive changes, known as “phase variation” where costly traits in different parts of a bacterial population can be activated/eliminated in an on/off fashion (Henderson et al., 1999; van den Broek et al., 2003; Vial et al., 2010). For example, it is believed that the frequent failure of “plant growth promoting bacteria” is due to the loss of plant protecting traits, like antifungal secondary metabolites, induced by these trade-offs (Chancey et al., 2002; van den Broek et al., 2003, 2005). Accordingly, bacterial traits may undergo rapid co-evolutionary changes during the lifetime of a growing plant root (Oger et al., 2004; Donn et al., 2015). Still, priority effects and the coverage of functional traits by beneficial rhizosphere bacteria are essential for the invasion resistance of the root microbiome to pathogens (Wei et al., 2015; Xiong et al., 2017).

## Plant Selection for Microbial Traits

Experimental evidence indicates that root tips are already actively involved in the selection of specific microorganisms. It has been shown that the polysaccharide composition of root mucilage selects for microorganisms with suitable glycosyl hydrolase composition (Amicucci et al., 2019), and further selection is evoked by extracellular DNA, antimicrobial proteins, and secondary metabolites (Hawes et al., 2012; Haichar et al., 2014). Some bacterial taxa, like Opitutaceae and members in Burkholderiales, might be especially adapted to the rapid assimilation of specific rhizodeposits in maize and other cereals (Hünninghaus et al., 2019; Nuccio et al., 2020). A mathematical model of bacterial growth dynamics after encountering a growing root tip by Dupuy and Silk (2016) identified early attachment to root tips as a decisive bacterial trait, because attached bacteria remaining on the maturing root gain access to larger quantities of exudate-C and can multiply at faster rates. Since bacterial colonization is limited by a finite number of attachment sites, Dupuy and Silk (2016) assumed that detachment and re-colonization of less populated root tip regions can substantially increase the microbial carrying capacity of roots, leading to a sharp bacterial peak density on tips. Microscopical studies of roots instead show a rather low and patchy colonization of root tips, and only as roots mature an increase in bacterial cell densities with more homogeneous distributions is observed (Schmidt and Eickhorst, 2014; Schmidt et al., 2018). On the other hand, typical rhizosphere microorganisms are often mobile, and bacteria, protists, and some rapidly growing fungi (e.g., “sugar fungi,” de Boer et al., 2006) move in parallel to the exudate pulse along the root (Hünninghaus et al., 2019). These organisms are generally characterized by high “rhizosphere competence,” being both, well adapted to fast colonization and fierce competitors for root colonization sites (Lugtenberg et al., 2001; Kamilova et al., 2005; Schreiter et al., 2014, 2018; Mulero-Aparicio et al., 2019).

The recruitment of microbes from the soil by plants is further influenced by the endogenous development of individual roots. The unequal distribution of microorganisms (Hebbar et al., 1992; Watt et al., 2006a; Buddrus-Schiemann et al., 2010) and their functional potential (Baudoin et al., 2002) between the actively growing root tip, root elongation, and maturation zones is associated with a spatial gene expression gradient along the root. This comprises a gradual transition from transcripts related to sugar-mediated signaling at the root meristematic zone to defense response related pathways toward the maturation zone (Hill et al., 2016; Stelpflug et al., 2016). Studies applying stable isotope probing (SIP) of plants and root-associated microorganisms indicate strong dynamics in the taxonomic composition and activity of microbial consumers in the rhizosphere (Drigo et al., 2010; Nuccio et al., 2020). For example, Hünninghaus et al. (2019) demonstrated that bacterial taxa with highest read abundance in the rhizosphere of maize were not necessarily those showing highest enrichment of ^13^C from rhizodeposition (Figure 1), indicating substantial differences in consumption of exudates. Similarly, Nuccio et al. (2020) reported a fast functional succession in carbohydrate depolymerization (CAZyme) genes in the rhizosphere of the grass *Avena fatua*, but functions of individual taxa were not mutually exclusive and only a handful of the bacterial taxa with CAZyme potential were actively involved metabolizing root carbon at any timepoint. Accordingly, active and passive exudation of low molecular weight carbon compounds, mostly sugars and organic acids, in the subsequent root expansion and root hair zones (Farrar et al., 2003) constitutes the next level of microbial community modulation (Hu et al., 2018). The root hair region was further shown to be a main regulator of rhizosphere enzyme activities (Giles et al., 2018; Zhang et al., 2020). Because root exudates contain a variety of plant species-specific metabolites and signal compounds with critical functional roles in plant defense and symbiosis (Baetz and Martinoia, 2014), they have been suggested to be the main drivers for the selection of root-specific microorganisms (Badri and Vivanco, 2009; Hartmann et al., 2009; van Dam and Bouwmeester, 2016; Hugoni et al., 2018; Nuccio et al., 2020).

## Circadian Rhythm and Microbiome Assembly

From a microbial perspective, however, deterministic community assembly processes are rather unlikely as long as resources are not limiting (Nemergut et al., 2013; Rüger et al., 2021). In maize, the abundant rhizodeposits of the root hair region even stimulate the production of bacterial extracellular polymeric substances and the formation of rhizosheaths as a mechanism to enhance root-soil contact (Watt et al., 1993, 1994). However, sugar metabolism in plants is subject to strong circadian regulation with the storage of photosynthates as starch during the day and its remobilization for growth and metabolism during the night, resulting in the depletion of carbohydrate reserves just before dawn (Bläsing et al., 2005; Stitt and Zeeman, 2012). These dynamics are reflected by pulsed exudation rates with strong diurnal fluctuations in the rhizosphere (Kuzyakov and Cheng, 2004). For example, in maize, exudation was found to be 1.5-fold reduced during night compared to daytime (Kuzyakov et al., 2003; Oburger et al., 2014). These transitory resource constraints in the rhizosphere have the potential to exert selection pressures on the functional traits and taxonomic composition of rhizosphere communities (Baraniya et al., 2018). The diurnal timing of rhizodeposition appears to be of major importance for understanding the assembly of microbiomes. Models and experiments by Semenov et al. (1999) and van Bruggen et al. (2008) indicate that the transient pulses of root exudates lead to a fast successions of competing microorganisms that may appear as moving waves along the root axis. Their models account for selection pressures that lead to oscillating dynamics between competing copiotrophic and oligotrophic bacterial taxa during root maturation, which are further enhanced by predator-prey dynamics of bacterivores (Zelenev et al., 2005, 2006).

## Predator-Prey Dynamics and Microbiome Assembly

There is increasing evidence that predator-prey dynamics of bacterivores contribute significantly to the deterministic processes that eventually determine microbiome composition and function (Rosenberg et al., 2009; Amacker et al., 2020; Rüger et al., 2021). Numbers of bacterivore protists show a steep increase toward the plant rhizosphere, where protist predation regulates bacterial turnover (Zwart et al., 1994; Alphei et al., 1996). Protist predation is known to rapidly alter bacterial community structure and function by changing bacterial competition and growth-defense trade-offs, e.g., due to upregulation of antibiotics production, and it further initiates microevolution of bacterial traits during the lifetime of a plant host (Young, 2006; Mazzola et al., 2009; Jousset and Bonkowski, 2010; Jousset, 2012; Friman et al., 2014; Song et al., 2015). The fact that the mere supernatants of protist cultures were sufficient to change the expression of bacterial defense-traits in culture (Jousset et al., 2010) demonstrates that studies focusing solely on bacteria-plant interactions fall short of what is required to understand the self-assembly of rhizosphere communities and microbiome functions. Few experiments to date studied how increased species richness of protistan predators feeds back on bacterial diversity and function. Single protist taxa differ in their feeding modes (Glücksman et al., 2010; Flues et al., 2017; Amacker et al., 2020), and higher species richness was shown to strongly increase the exploitation of bacterial prey species (Saleem et al., 2012, 2013; Hünninghaus et al., 2017). It appears that protistan predation is enhancing bacterial biocontrol activity on roots (Weidner et al., 2016; Xiong et al., 2018; Gao et al., 2019; Amacker et al., 2020). Resulting changes in bacterial community composition apparently feedback again on the composition of communities of bacterivore rhizosphere protists (Sapp et al., 2017; Rossmann et al., 2020). Accordingly, we hypothesize that the self-organization of bacterial rhizosphere communities is shaped by both bottom-up processes *via* resource supply from rhizodeposits and top-down processes through grazing activities by protists, or even predatory bacteria and bacteriophage viruses (Geisen et al., 2017; Vetterlein et al., 2020). In other words, we believe that the net impact between predators and prey is driven by reciprocal selection through a coevolutionary arms race toward grazing-resistant bacteria and rapid adaptation of protistan grazers. Focusing only on bottom-up processes will miss a crucial feedback mechanism on bacterial functioning.

## Influence of Plant Growth Stage on Microbiome Assembly

On longer time scales, microbiome composition changes as plants mature. Root growth dynamics and vitality of roots change dynamically during the lifetime of a maize plant (Fusseder, 1987; Pagès and Pellerin, 1994; Pellerin and Pagès, 1994). Simultaneously, the contributions of different root types to root architecture are changing (Wang et al., 1994; Hochholdinger, 2009; Yu et al., 2018). The resulting associated alterations in the quantity and composition of root exudates during plant development directly feed back on microbiome composition (Aulakh et al., 2001; Chaparro et al., 2013). The consequences of these phenological changes on species turnover of microbiomes and changes in regulatory microbiome functions are as yet little understood. Field studies indicate that the compostition of bacterial and fungal communities is more significantly influenced by plant growth stages than by fertilization level, and that the most temporally separated stages show the most significant differences in both bacterial and fungal communities (Wang et al., 2016, 2018). For instance, Ascomycota are enriched during fast seedling growth rate, but Basidiomycota and Glomeromycota are more abundant later, during flowering. These changes have functional relevance, since community-level physiological profiling demonstrated that the rhizosphere communities of different plant species were more similar at the same than at different developmental stages (Houlden et al., 2008). Plant rhizodeposits are differentially produced at distinct stages of development, and Chaparro et al. (2014) related such changes to differential distribution of bacterial phyla and representation of their traits in an experimental culture system. Acidobacteria, Actinobacteria, Bacteroidetes, and Cyanobacteria followed distinct patterns associated with plant development and root exudation, and metatranscriptomics analysis of the rhizosphere microbiome revealed changes in, for instance, genes involved in antibiosis. From root exudates, phenolic compounds appeared to mediate changes in both the relative 16S rRNA and the transcript abundances (Chaparro et al., 2014). In the field, factors that co-vary with plant growth stages include in particular soil temperature, and soil moisture, as well as crop growth rate. It appears that plants reduce root exudation and investments into microbiome functioning when plant physiology changes from vegetative to reproductive growth (Aulakh et al., 2001; De-la-Peña et al., 2010). This is generally accompanied by a reduction of species richness and changing abundances of rhizosphere microbiota (Toljander et al., 2008; Chaparro et al., 2014; Schmidt and Eickhorst, 2014). In this respect, the modern “stay-green” hybrids of maize, characterized by a delayed canopy senescence, deserve particular attention (Ding et al., 2005; Lee and Tollenaar, 2007), because their longer photosynthetic activity and higher N-demand together with increased total root length and deeper roots compared to older varieties (Echarte et al., 2008; Ning et al., 2014; Antonietta et al., 2016) likely feeds back on microbiome persistence and functioning. Taken together, shifts in the spatio-temporal patterns of microbial abundance and community composition at different developmental stages could impact microbiome functions at larger scales, such as decomposition and nutrient mobilization processes. However, the effects of phenological changes on the composition of the maize microbiome are currently too poorly understood to allow more exact predictions of its functional implications.

## Outlook and Application

Currently, network analysis appears as the most promising tool to identify relevant patterns in the trophic structure and complex co-occurrences of taxa among multiple domains in the microbioime, i.e., bacteria, archaea, fungi, and protists (Rüger et al., 2021); and network analysis allows to integrate further multi-omics data (e.g., root exudate profiles, proteomics, peptidomics, and gene expression) and their correlation to specific taxa (for current approaches, see reviews by Jiang et al., 2019; Trivedi et al., 2020). For practical applications, the aim must be to identify microbial consortia affecting specific plant traits (Vílchez et al., 2016; Oyserman et al., 2018), then strong and weak phenotypic responders among plant varieties could be identified by bulk segregant analysis, and resulting inbred lines after crossing a strong and weak responder cultivar can be used to identify plant molecular control points through quantitative trait loci (Quarrie et al., 1999; Phillips and Strong, 2003). Current plant microbiome studies are often still rather descriptive, but the tools are available to approach hypotheses-driven, targeted manipulations of plant microbiomes to improve plant breeding for a more sustainable agriculture (Wei and Jousset, 2017; Jacoby et al., 2021).

## Summary

The microbial community assembly in the rhizosphere is as dynamic as root growth with distinct diurnal and phenological changes during the lifetime of the plant. The assembly of the rhizosphere microbiome along the root axis is initiated at the root cap with a significant community shift from metabolically inactive to fast-growing, copiotrophic taxa. Apparently even at this early stage of root growth, both plant secondary metabolites and bacterial traits of “rhizosphere competence” contribute to the preference of particular taxa in the microbiome. Still, a crucial prerequisite for the operation of deterministic assembly processes is the constraint of resources. Therefore, the abundant resource supply from rhizodeposition likely counteracts microbiome selection and favors stochastic (i.e., “neutral”) processes of community assembly, especially through priority effects at initial stages of root colonization. Accordingly, the exploitation of priority effects appears most promising for the targeted optimization of microbial consortia for microbiome engineering. At some point, the initially random, neutral assembly of free-living microbiota will be replaced by increasingly deterministic assembly processes; reflected by low variability of beta diversity between communities. Deterministic processes of community assembly become more likely when the resources from rhizodeposits subside and roots mature. A subsequently increased competition for resources in concert with top-down control by predators, may eventually lead to the formation of a stable rhizosphere “microbiome.” Still, there are a number of unresolved issues. Diurnal fluctuations in magnitude and composition of rhizodeposits may play a more important role for microbiome assembly than currently appreciated. Different root types of maize bear different microbial communities and it would be interesting to know whether the enhanced variability of microbial taxa also covers broader functional versatility. In particular, uncovering the spatial and temporal coordination of microbiome composition and function during the development of plants remains a challenging task in future studies.

## Author Contributions

MB drafted the manuscript. All authors contributed significantly to improve the manuscript by intensive discussions and substantial additions and revisions of text passages.

### Conflict of Interest

The authors declare that the research was conducted in the absence of any commercial or financial relationships that could be construed as a potential conflict of interest.
